# Type I interferon signaling and macrophages: a double-edged sword?

**DOI:** 10.1038/s41423-020-00609-0

**Published:** 2021-01-08

**Authors:** Barbara Adler, Heiko Adler

**Affiliations:** 1 Max von Pettenkofer Institute & Gene Center, Virology, Faculty of Medicine, LMU München, Munich, 81377 Germany; 2 Research Unit Lung Repair and Regeneration, Comprehensive Pneumology Center, Helmholtz Zentrum München - German Research Center for Environmental Health (GmbH), Member of the German Center of Lung Research (DZL), Munich, Germany

**Keywords:** Infection, Infectious diseases

In a recent issue of the Journal of Experimental Medicine, Zhang et al. reported that type I interferon (type I IFN) signaling mediates Mycobacterium tuberculosis (Mtb)-induced macrophage (MΦ) death, most likely by a new, currently unknown cell death pathway.^1^

**Fig. 1:**
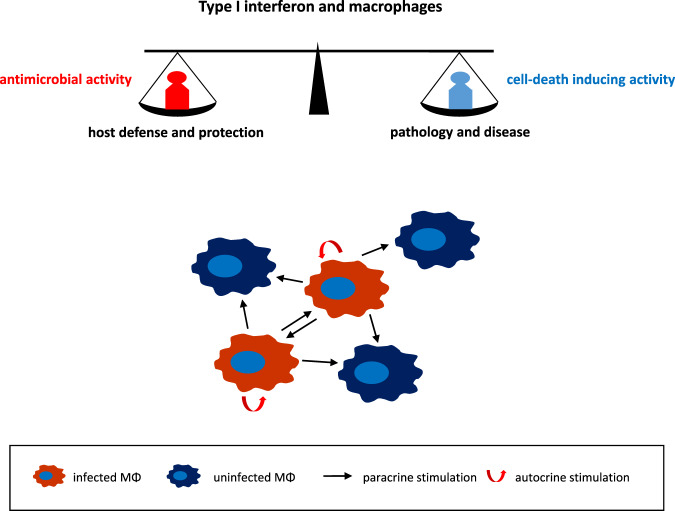
Infected macrophages produce type I interferon that by autocrine and paracrine signaling may exert antimicrobial effects, contributing to host defense and protection, or induce macrophage death, potentially leading to pathology and disease. The final outcome is most likely context-dependent, i.e., determined by the pathogen type, infection dose, host genetics, and target organ

Type I IFNs are a major host defense against viral and bacterial infections. They are produced by a variety of cell types, including MΦs. After recognition of pathogen-associated molecular patterns by, for example, Toll-like receptors, host cells produce type I IFNs that can act in both autocrine and paracrine ways to activate or repress IFN-stimulated genes.^2,3^ In addition to interfering with multiple stages in the life cycle of pathogens, type I IFNs have additional functions influencing both innate and adaptive immune responses, which can result in beneficial but also detrimental effects in the host (Fig. 1). It seems that the outcome of the type I IFN response is highly context-dependent.^3^ For example, type I IFN signaling during bacterial infections is dependent on many factors, e.g., whether the bacteria are intra- or extracellular, thereby activating different signaling pathways. While type I IFN signaling is crucial for host defense against some bacteria, e.g., pneumococci, it may promote infection by others, including Mtb.^3,4^ Mtb infects MΦs, and once inside the cell, it inhibits the development of phagosomes to phagolysosomes, enabling Mtb not only to survive but also to replicate. Infected MΦs will eventually die, releasing bacteria that spread to more cells. Thus, Mtb-induced MΦ death is a crucial factor in the pathogenesis of tuberculosis. Despite the importance of Mtb-induced MΦ death, the underlying mechanism remains elusive.

Therefore, Zhang et al.^1^ investigated which type of cell death Mtb-infected MΦs undergo. Surprisingly, they discovered that the hitherto known types of cell death are obviously not involved. Specifically, by using a variety of approaches, e.g., the application of inhibitors of specific cell death pathways and knockout cells, they excluded apoptosis, pyroptosis, necroptosis, parthanatos, ferroptosis, and autophagy-dependent cell death. From these data, the authors concluded that Mtb-induced MΦ death might involve a novel mechanism. To identify this mechanism, the authors performed a genome-wide CRISPR-Cas9 screen in an Mtb-infected MΦ cell line. This screen identified type I IFN signaling as important for the death of Mtb-infected MΦs. Subsequent genetic and immunological studies confirmed that autocrine and paracrine type I IFN signaling play an important role in the death of Mtb-infected MΦs. Crucially, the authors went on to demonstrate that blocking type I IFN signaling protects Mtb-infected mice and, importantly, augments the benefit of rifampin, a medication used to treat drug-sensitive tuberculosis.

For tuberculosis, the connection that type I IFN signaling is detrimental to the host by promoting MΦ death and propagation of Mtb from dead MΦs is new. For *Salmonella* typhimurium, type I IFN signaling has already been implicated in MΦ death during infection.^5^ In this case, it induces MΦ necroptosis, resulting in reduced control of infection. During Listeria infection, type I IFN promotes apoptotic MΦ cell death, leading to innate immune suppression.^6^ Type I IFN-induced MΦ cell death must not necessarily be direct. More than two decades ago, we described that IFN-α can prime macrophages for activation-induced apoptosis.^7^ Exposure of MΦs to recombinant or herpesvirus-induced IFN-α followed by activation with lipopolysaccharide (LPS) induced MΦ apoptosis. We suggested that this pathway might contribute to the pathogenesis of diseases. Indeed, we found that MΦs infected with the cytopathic biotype of bovine viral diarrhea virus (BVDV) produced factors, including type I IFN, that primed both infected and uninfected MΦs for LPS-induced apoptosis. Considering that the principal lesions of mucosal disease, the lethal form of infection with cytopathic BVDV, are located in regions with high concentrations of endotoxin (the oral cavity and gastrointestinal tract), our findings strongly suggested a role of this pathway in the pathogenesis of mucosal disease.^8^

By showing a key role for type I IFN signaling in Mtb-induced MΦ cell death, Zhang et al.^1^ uncovered a new mechanism for the adverse effects of pathogen-induced type I IFN on disease outcome. Furthermore, they suggest that MΦ cell death occurs via a new, yet unknown, cell death pathway. Type I IFN-dependent MΦ cell death thus results in the release of bacteria from infected MΦs, securing Mtb spread, and very likely in the release of factors that trigger inflammation and tissue damage. The latter might be particularly relevant in the lung, where a balance between protective and pathological immune responses is highly important to minimize immunopathology and maintain pulmonary function.^9^ The findings of Zhang et al. suggest that blockade of type I IFN signaling might be a new therapeutic avenue for the treatment of tuberculosis. In particular, and as they already showed in a mouse model, blocking type I IFN signaling could perhaps be applied in addition to treatment with antibiotics. Thus, the development of new treatment strategies will be a future research path. In addition, the new, unknown cell death pathway and its underlying mechanism warrant further study. Overall, the findings of Zhang et al. may be generally important not only for tuberculosis but also for the pathogenesis of bacterial and perhaps viral infections. Elucidating the details of type I IFN signaling in MΦ cell death might lead to new host-directed therapies for a variety of diseases.
